# Activation of BK channels ameliorates cardiac injury Via NFκB-NLRP3 signaling in angiotensin II-induced hypertension mouse model

**DOI:** 10.3389/fphar.2026.1739677

**Published:** 2026-06-15

**Authors:** Long Zhao, Xingchen Wu, Xiaomao Luo, Anwen Zheng, Qiang Yue, Haidong Wang, Junko Ishida, Cesar A. Romero, Douglas C. Eaton, Janet D. Klein, Xiaonan H. Wang, Yan Xu, Hui Cai

**Affiliations:** 1 Renal Division, Department of Medicine, Emory University, Atlanta, GA, United States; 2 Department of Nephrology, The Affiliated Hospital of Qingdao University, Qingdao, China; 3 College of Veterinary Medicine, Shanxi Agricultural University, Taigu, Shanxi, China; 4 Emory College of Arts and Sciences, Emory University, Atlanta, GA, United States; 5 Department of Food and Nutritional Environment, College of Human Life and Environment, Kinjo Gakuin University, Nagoya, Japan; 6 Nephrology Section, Atlanta VA Medical Center, Decatur, GA, United States

**Keywords:** cardiac fibrosis, cardiovascular disease, hypertrophy, NFκB, ROS, SOD

## Abstract

**Background:**

Hypertension-induced cardiac fibrosis leads to heart failure. Large-conductance calcium-activated potassium (BK) channels regulate vascular tone, and their activation may mitigate fibrosis. This study explores the impact of BK channel activation on angiotensin II (Ang II)-induced hypertension and cardiac fibrosis and its effects on nuclear factor-kappa B (NFκB) signaling.

**Methods:**

Male C57BL/6 J mice were infused with Ang II for 4 weeks to induce hypertension and cardiac fibrosis. The BK channel activator BMS-191011 was administered via intraperitoneal injection. Cardiac tissue was analyzed using histology, Western blotting and qPCR. Single channel recordings were used to analyze the activation of BKα channels. A dihydroethidium (DHE) Assay was used to detect superoxide (ROS) production. Superoxide Dismutase (SOD) was measured using a colorimetric activity kit. *In vitro*, macrophages (RAW 264.7) were stimulated with Ang II, to detect NFκB activation and macrophage polarization.

**Results:**

The expression of BKα mRNA and protein abundance was decreased while markers of cardiac fibrosis (fibronectin, vimentin, αSMA and TGF-β) and ROS production were increased in the hearts of Ang II-induced hypertensive mice. In addition, macrophage infiltration was significantly increased along with inflammatory cytokines IFNγ, IL-6, IL-4, IL-10 and TNFα. The BK channel opener, BMS-191011, significantly reduced hypertension, cardiac fibrosis, and ROS production, but restored SOD in the hearts of Ang II-treated mice. In cultured macrophages, we found that Ang II increased inflammatory cytokines through NFκB-NLRP3-caspase-1 signaling. After Ang II treatment, BK channel activation inhibited NFκB, NLRP3 and caspase-1 expression. BK channel activation also suppressed NFκB-NLRP3 signaling, leading to reduced ROS accumulation and restored SOD activity.

**Conclusion:**

BK channel activation reduces Ang II-induced hypertension and cardiac fibrosis by modulating oxidative stress, inflammation and macrophage polarization through the NFκB-NLRP3 pathway. These findings implicate BK channel activators as a potential novel therapeutic strategy for hypertensive cardiomyopathy.

## Graphical Abstract

**Figure:**
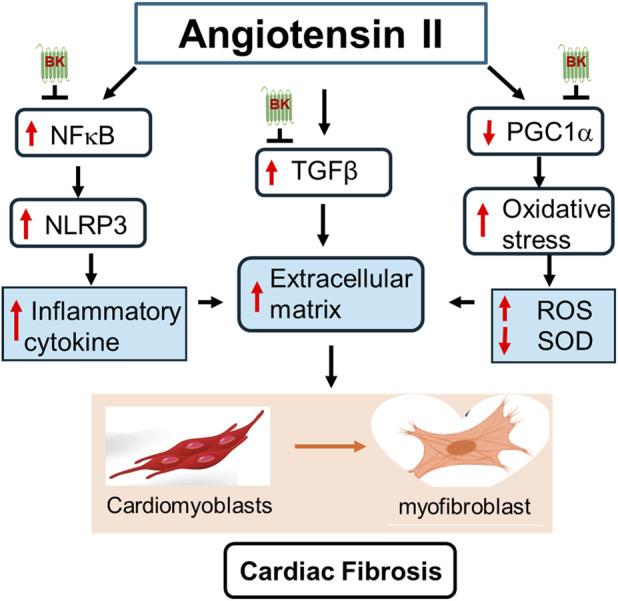
This study illustrates how BK channel activation as a potential therapeutic strategy that links redox balance, metabolic regulation, and inflammation control in cardiac fibrosis. Angiotensin II overactivation enhances NF-κB signaling, boosting inflammatory cytokines, and promotes TGF-β–mediated extracellular matrix buildup. At the same time, Angiotensin II reduced PGC1α lowers antioxidant defenses, increasing ROS and decreasing SOD, which amplifies oxidative stress and leading to promote the production of cytokines and growth factors, such as TGF-β. Additional, Angiotensin II has potensial to directly increase TGF-β–mediated extracellular matrix accumulation. Together, these pathways heightened inflammation, impaired redox balance, and excessive matrix deposition, leading to promote cardiomyoblast-to-myofibroblast transition, and pathological remodeling of the heart. Activation of BK channels, in parallel, act as a critical brake on the shift of cardiomyoblast into myofibroblast, and adverse cardiac remodeling by inhibiting inflammation, oxidative stress, and fibrotic remodeling.

## Introduction

Hypertension remains a pervasive public health challenge and a major contributor to cardiovascular diseases, which collectively account for substantial morbidity and mortality worldwide (Whelton et al., 2018). Despite advances in treatment strategies, the intricate mechanisms underlying hypertension and its associated cardiac damage continue to evade complete understanding. Ion channels, transcriptional factors, and signaling cascades are recognized as central regulators of vascular stability and the preservation of organ function (Ma et al., 2023). This study explores the protective role of large-conductance calcium-activated potassium channel (BK) activation in ameliorating angiotensin II (Ang II)-induced hypertension and associated cardiac fibrosis by targeting NFκB signaling.

Cardiac fibrosis is a pathological process characterized by excessive deposition of extracellular matrix (ECM) proteins, which disrupts normal myocardial architecture and impairs cardiac function. Hypertension-induced cardiac fibrosis poses significant challenges in clinical management due to its progressive and irreversible nature (Weber et al., 2013). Understanding the molecular mechanisms underlying cardiac fibrosis is crucial for developing novel therapeutic strategies.

Ang II, a key effector of the renin-angiotensin-aldosterone system (RAAS), exerts potent effects on vascular tone, sodium retention, and fluid balance. Elevated levels of Ang II are a hallmark of hypertension and contribute significantly to the development of cardiac fibrosis (Yao et al., 2022). Ang II induces fibrosis mediated by transforming growth factor-β (TGF-β) signaling, which drives the differentiation of fibroblasts into myofibroblasts, enhancing ECM deposition and tissue stiffening (Leask, 2007). This process culminates in myocardial fibrosis, impaired diastolic function, and heart failure. However, whether BK channel activation can attenuate Ang II-induced cardiac fibrosis remains unclear, and more importantly, there is a lack of comprehensive mechanistic studies linking BK channel activation to the inflammatory–fibrotic axis, particularly the NF-κB–NLRP3 signaling pathway, in hypertensive cardiac injury.

BK channels are abundantly expressed in vascular smooth muscle cells, renal tubules, and cardiac myocytes where they regulate membrane potential and cellular excitability (Pantazis and Olcese, 2016). BK channel activation facilitates potassium efflux, leading to hyperpolarization of the cell membrane and relaxation of vascular smooth muscle (Dopico et al., 2018). By promoting vasodilation, BK channel activation reduces systemic vascular resistance and lowers blood pressure (Wang et al., 2022a). Recent evidence suggests that BK channel activity extends beyond blood pressure regulation, influencing processes such as oxidative stress, inflammation, and fibrosis (Wang et al., 2022b). Activation of BK channels has been associated with reduction of myocardial hypertrophy and interstitial fibrosis by suppressing pro-fibrotic signaling pathways (Xing et al., 2023). These findings underscore the potential of BK channels as therapeutic targets in hypertension and related cardiac complications.

The diseased myocardium forms a hostile microenvironment characterized by inflammation, oxidative stress, and matrix disruption, which severely impairs tissue repair and limits therapeutic efficacy (Yao et al., 2026). Nuclear factor-kappa B (NFκB) is a pivotal transcription factor that orchestrates inflammatory and stress responses in cardiovascular diseases (Zhu et al., 2023). Persistent NFκB activation in cardiomyocytes and fibroblasts promotes extracellular matrix deposition and maladaptive ventricular remodeling (Ren et al., 2021), and, as expected, Pharmacological or genetic inhibition of NFκB signaling effectively attenuates myocardial inflammation, reduces fibrosis, and preserves cardiac function in experimental models (Ye et al., 2023). Moreover, NFκB integrates with parallel pathways such as MAPK and Akt/ERK, amplifying Ang II-mediated injury and remodeling (Shen et al., 2023; Gong et al., 2021). Recent studies also indicate that NF-κB activation in macrophages contributes to immune dysregulation and exacerbates hypertensive cardiac injury (Qi et al., 2014; Feng et al., 2022). Collectively, these findings highlight NFκB as a central mediator bridging hemodynamic stress and inflammatory fibrosis so targeting this pathway may offer a promising therapeutic strategy for hypertensive heart disease.

Given the interplay between BK channels, NFκB signaling, and Ang II-induced hypertension, this study aims to elucidate the therapeutic potential of BK channel activation in mitigating hypertension and cardiac fibrosis. Specifically, we hypothesize that BK channel activation attenuates NFκB-mediated inflammation and pro-fibrosis, thereby improving cardiovascular outcomes. To test this hypothesis, we investigated the effects of pharmacological BK channel activation in a mouse model of Ang II-induced hypertension and cardiac fibrosis, focusing on blood pressure regulation, fibrotic markers, oxidative stress, inflammatory and NFκB signaling pathways. By advancing our understanding of the impact of BK channel on the NFκB-NLRP3 axis, this research seeks to identify novel therapeutic strategies for hypertension and its associated cardiac complications.

## Methods

### Animals and treatments

The animal protocols were approved by the Emory Institutional Animal Care and Use Committee and adhere to NIH standards for animal use. Male C57BL/6 J mice were purchased from Jackson Laboratory (Bar Harbor, ME). All mice were maintained at 19 °C–21 °C on a 12-h/12-h light-dark cycle, were fed a standard rodent diet and allowed free access to drinking water. For Ang II hypertension model, micro-osmotic pumps were implanted subcutaneously in the mid-scapular region of male mice (8 weeks old). The osmotic pumps delivered Ang II (A2900 Sigma-Aldrich, MO) at a rate of 1.5 mg/kg per day for 4 weeks. BMS-191011 (SML0866, Sigma-Aldrich, MO) was administered at a dose of 20 mg/kg BW/day by IP injection daily for 4 weeks beginning from the day of surgery, a regimen selected based on previous *in vivo* studies demonstrating effective BK channel activation and anti-fibrotic effects at similar doses (Ning et al., 2022). Blood pressure was measured by tail-cuff with a Visitech Systems, BP-2000 Series II Blood Pressure analysis system (model BP-2000-CU with platform model BP-2000-MP-6), as previously described (Verlander et al., 2011). The number of animals used in each group was N = 4–5, as also indicated in the corresponding figure legends.

### Western blot and antibodies

Cardiac tissue or cells were homogenized by Polytron (PT45-80GT, Kinematica, Inc. Bohemia, NYin Gentle Lysis Buffer (10 mM Tris-HCl, 10 mM NaCl, 2 mM EDTA, 0.5% NP-40, 1% glycerol, and freshly added: 1 mM Na3VO4; 10 μg/mL PMSF; 5 μg/mL Aprotinin; 1 μg/mL Leupeptin) with phosphatase inhibitors cocktail one and 2 (P5726 Sigma). Proteins (30 μg/lane) were separated by SDS-PAGE and Western blotting was performed as previously described (Efe et al., 2016). Protein bands were scanned and quantified using the Li-cor Odyssey infrared scanning system (Li-COR Biosciences, Lincoin, Nebraska). Protein band intensities were normalized to GAPDH as an internal loading control and quantified using the Li-COR Odyssey infrared scanning system and ImageJ (NIH). Antibody for BKα (Apc-021) was from Alomone Labs (Jerusalem, Israel). Fibronectin (F3648), α-SMA (A2547) and Vimentin (V6630) antibodies were from Sigma-Aldrich. NLRP3 (R4775) was from VWR (Radnor, PA). Caspase 1 (MA16215) and pNFκBp65 (MA5-15160) were from Fisher Scientific (Norcross, GA). NFkBp65 (ab16502), PGC1α (AB3242) and TGFβ (ab215715) were from Abcam (Cambridge, MA). TNFα (SC-133192) and 1L-1bβ (SC-7884) were from Santa Cruz Biotechnology (Santa Cruz, California). The antibody for GAPDH (MAB 374) was from Millipore (Burlington, MA).

### Immunohistochemistry and histology

Hearts in paraffin sections were deparaffinized and hydrated, then fixed with 4% paraformaldehyde and immunohistochemically stained as previously described (Efe et al., 2016). Masson’s Trichrome staining was performed following the manufacturer’s recommended protocol (IMEB Inc., San Marcos, CA). Mac-2 staining was performed by an ImmPRESS Polymerized Reporter Enzyme Staining System (vector laboratories; MP-6402-15). The Samples were blocked with 2.5% horse serum for 1 h, followed by incubation overnight with anti-Mac-2 antibody (Santa Cruz, CA). Sections were further incubated for 60 min with ImmPRESS®-VR Horse Anti-Mouse IgG for 30 min, and peroxidase substrate solution, (VectorLabs, Newark, CA). The Images were visualized with an Olympus inverted microscope IX71 with CellSens software (Olympus, Melville, NY, USA). Quantitative evaluation was performed by computerized analysis using ImageJ (NIH).

### Quantitative RT-qPCR analysis of mRNA

Total RNA was extracted using Tri-Reagent (Molecular Research). RNA was reverse transcribed to synthesize cDNA using M-MLV Reverse Transcriptase (Invitrogen). Designed primers are listed in Supplementary Table S1. Expression of individual mRNA was standardized to 18s gene expression and calculated as the difference between the threshold values of two genes (ΔΔcq) (Wang et al., 2017).

### Reactive oxygen species (ROS) and superoxide dismutase (SOD) detection

Intracellular level of ROS was measured in mice heart tissue using Reactive Oxygen Species Detection Assay Kit (Abcam ab287839). Fluorescence was detected at Ex/Em = 495/529 nm in presence of experimental compounds and controls. Fluorescence Microplate reader (Biotek Synergy 2 Plate Reader Multi-Mode) was used for this analysis. Fluorescence was detected at excitation 560 nm and emission detection at 590 nm. Activity was established as the change in fluorescence after background subtraction. SOD activity was detected by SOD Colorimetric Activity Kit (Invitrogen ELASODC MAN0019052). Samples and standard were read at the absorbance at 450 nm. The background absorbance was subtracted from all data points, including standards.

### Cell culture

HEK293/BKα cells: The cells stably over-expressing BKα were generated in our lab as previously reported (Liu et al., 2015). Cultured cells were maintained in DMEM supplied with penicillin (100 U/mL), streptomycin (100 μg/mL), Geneticin Selective Antibiotic (G418 Sulfate) at 200 μg/10 mL, and 10% FBS. G418 was removed when cells were prepared for experiments.

Macrophages (RAW 264.7 TIB-71, ATCC) were grown in Dulbecco’s Modified Eagle’s Medium (DMEM) with 10% FBS, 100 U/mL penicillin, 100 μg/mL streptomycin, and 4 mM L-glutamine. The BK channel opener NS-1619 (Sigma-Aldrich, St. Louis, MO, USA; Cat# N170-25 MG) was dissolved in DMSO to prepare a stock solution and diluted in culture medium to the indicated final concentrations (20 μM) immediately before use.

### Single-channel recording in cardiac myoblasts

All single channel experiments in this study were carried out using the cell-attached configuration of patch-clamp in HEK293/Bkα cells. The bath solution used in the cell-attached mode contained the following (in mM): 140 NaCl, 10 CaCl2, 10 KCl, 1 MgCl2, and 10 HEPES, pH adjusted to 7.4. Single-channel patch-clamp was performed as previously described (Liu et al., 2015). In prior work, calcium was elevated slightly (2–3 mM to 10 mM) and potassium was also elevated (3.5–4.5–10 mM) to increase the frequency of BK channel opening and to stabilize membrane potential. Briefly, a microelectrode (pipette) was filled with pipette solution (140 NaCl, 10 CaCl2, 10 KCl, 1 MgCl2, and 10 HEPES, pH 7.4) and lowered to a single cell before application of a small suction to achieve a >1 Gohm seal. The channel currents were recorded with an AxoPatch 1D amplifier (Molecular Devices) sampled at 10 kHz and filtered at 5 kHz. The patch was held at a pipette potential of −100 mV and recorded from continuously for a mean time of 4.6 ± 1.01 min and a mean number of 5,954 events per patch. Channel activity (NPo) was calculated from pClampfit 10.7 data analysis software (Molecular Devices). Channel number (N) was estimated from the maximal number of transitions during total recording time, and channel Po was calculated as the ratio of NPo to N.

### Statistical analysis

All data are presented as mean ± SEM. SAS (Statistical Analysis System) software was used for statistics. A 2-tailed Student’s t*-*test was used for comparisons of two groups. For a comparison of more than two groups, we used one-way ANOVA with a *post hoc* analysis by Bonferroni test. To analyze an interaction between two factors, we used two-way ANOVA, repeated measures with Tukey as the post-test. Chi-squared test was used to analyze the fraction of patches with BK channels. Statistical differences with *P* < 0.05 were considered significant.

## Results

### BK protein decreases in the hearts of ang II-induced hypertensive mice, but activation of BK channels reduces ang II-induced hypertension

**FIGURE 1:**
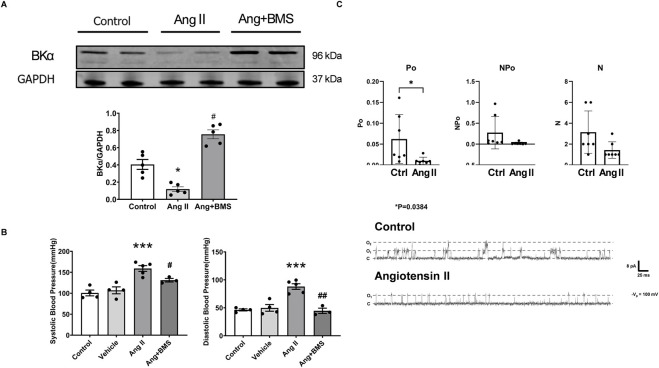
BK protein decreased in the hearts of mice with Ang II-induced hypertension. Activation of BK channels reduced Ang II-induced hypertension. **(A)** Western blot of lysates from the mouse hearts of control, Ang II, and Ang II + BMS191011 probed for BK channel proteins. Protein abundance was normalized to GAPDH. Quantification is shown as mean ± SEM (N = 5). *P < 0.05 vs. control and #P < 0.05 vs. Ang II by two-way ANOVA. **(B)** Systolic and diastolic blood pressures were measured in control, vehicle, Ang II and Ang II plus BMS191011-treated mice. All data: Mean ± SEM (N = 3–5). ***P < 0.001 vs. control and *P < 0.05, **P < 0.01, vs. Ang II by two-way ANOVA. **(C)** BK channel activity was recorded from cell-attached single-channel patches on HEK 293 cells expressing BKα, following treatment with Ang II (100 nM) for 24 h. Representative traces show BK channel openings (o1, o2) and closed state **(C)**. Recordings were obtained at a pipette holding potential (Vp) of 100 mV. Dotted lines indicate channel open and closed levels. Channel activity was quantified as open probability (Po), product of number of channels and open probability (NPo), and number of channels (N). Ang II treatment significantly reduced Po compared to control (*P = 0.0384 b y unpaired two-tailed Student’s t-test), while NPo and N showed no significant change. Data are presented as mean ± SEM (N = 7 per group).

A prior study shows that angiotensin II has an inhibitory effect on Bk channel activation (Yin et al., 2019). To identify whether the renin-angiotensin system impacts BK channels, Ang II, administered by osmotic minipumps for 4 weeks (1.5 mg/kg BW/day), was used to induce hypertension in the C57BL6 mice. BMS-191011 (BMS 20 mg/kg BW/day), a BK channel opener, was intraperitoneally injected (IP) into mice every day to activate BK channels. Mice were randomly assigned to control, vehicle (0.15% DMSO +0.05% Emulsifier +99.8% normal saline), Ang II, or Ang II + BMS groups. Blood pressure was measured before, after 2-week and at the end of the experiment. The heart was harvested after 4 weeks. Western blots showed that the amount of BKα protein was significantly decreased in the Ang II cohort compared to the controls (Figure 1A). Conversely, the level of BKα protein was increased in the Ang II + BMS group compared with the Ang II group. Both systolic blood pressure (BP) and diastolic BP at harvest was significantly increased in the Ang II cohort compared with the control mice, whereas intervention with BK opener abolished the Ang II-induced hypertension (Figure 1B).

To explore whether the Ang II effects BKα activity, cultured HEK/BKα cells (stable BKα overexpressing cell line) were treated with Ang II (100 nM) followed by single-channel recording. The fraction of patches with BK channels in untreated control cells was 0.27 (7 out of 26) patches) and in Ang II treated cells the fraction was 0.019 (7 out of 37 patches). A Chi-squared test suggests that Ang II significantly decreased BKα activity measured as open probability compared to channels in untreated cells (Figure 1C). The open probability (Po) for untreated patches with channel activity was 0.0621 ± 0.0588 (n = 7); and the Po for patches with activity after treatment with ANG II was 0.00984 ± 0.00869 (n = 7). These results suggest that Ang II decreased BKα protein amount and reduced BK channel open probability in the hearts of hypertensive animals. Administration of BKα opener limited Ang II-induced hypertension and restored BKα protein to control levels.

### Activation of BKα attenuated ang II-induced cardiac fibrosis and downregulated TGF-β in mice

**FIGURE 2:**
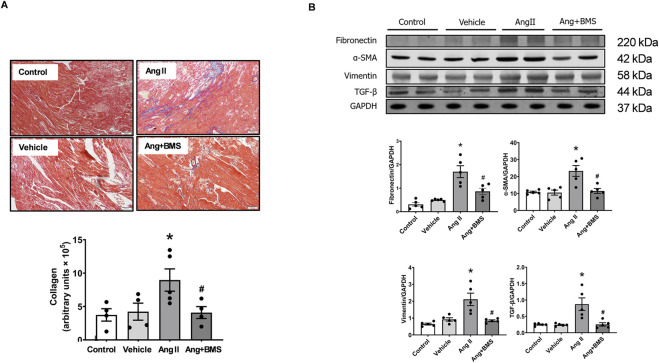
Activation of BKα attenuated Ang II-induced cardiac fibrosis and downregulated TGF-β in mice. **(A)** Masson’s Trichrome staining was performed on heart tissues from in control, vehicle, Ang II, and Ang II plus BMS191011-treated mice. Fibrotic tissue (collagen) is shown in blue. Images were captured at 200× magnification; scale bars = 50 μm. Quantification of fibrosis was calculated as the percentage of fibrotic area over the total tissue area using ImageJ analysis software. Data: mean ± SEM (N = 4–5). *P < 0.05 vs. control and #P < 0.05 vs. Ang II by two-way ANOVA. **(B)** Protein abundance of fibronectin, αSMA, vimentin and TGF-β were determined by Western blot in the heart of mice across treatment groups. All data was normalized to GAPDH as control. Mean ± SEM (N = 5). *P < 0.05 vs. control and #P < 0.05 vs. Ang II by two-way ANOVA.

Our previous studies showed that BK activation has an anti-fibrotic effect on kidney fibrosis (Wang et al., 2022b). To discover whether a BK opener could block fibrosis in the heart, Masson’s trichrome staining was used to determine fibrosis area in the heart tissue of Ang II treated mice. The percentage area of collagen deposits (indicated by blue staining in heart sections) areas was sharply increased by 2.5-fold in the Ang II cohort vs. controls, while intervention with BMS191011 reduced the elevated collagen accumulation by 54% compared with the Ang II group (Figure 2A). Three fibrosis-induced proteins were examined to evaluate the effects of BKα activation on Ang II–induced cardiac fibrosis. Fibronectin, a key interstitial matrix protein, serves as a signature marker for myofibroblast activation. Alpha-smooth muscle actin (α-SMA) is commonly used to identify myofibroblast formation, while vimentin marks cells undergoing epithelial-to-mesenchymal transition. All three markers were significantly increased in the hearts of Ang II–treated mice. Administration of the BK channel activator BMS-191011 reduced the expression of these fibrotic markers in cardiac tissue (Figure 2B). Additionally, BMS-191011 administration reduced the Ang II–induced increase in cardiac TGFβ protein levels. These findings indicate that BKα activation reduces cardiac fibrosis associated with the increase of TGFβ signaling in Ang II–induced hypertensive mice.

### Activation of BKα eliminated ang II-induced upregulation of oxidative stress in the cardiac tissues of mice

**FIGURE 3:**
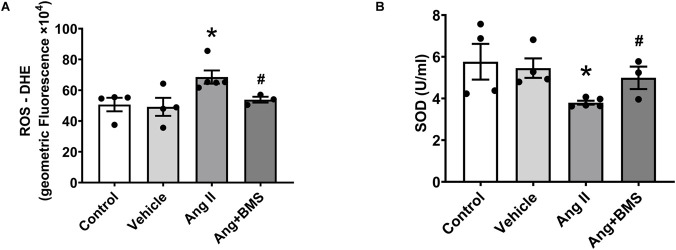
Activation of BKα eliminated Ang II-induced upregulation of oxidative stress in the cardiac tissues of mice. **(A)** Intracellular levels of ROS production were measured in control, vehicle, Ang II and Ang II plus BMS191011-treated mice. Absorbance (Ex495/Em529) was identified in a fluorescence microplate reader. All data: mean ± SEM (N = 3–5). *P < 0.05 vs. control and #P < 0.05 vs. Ang II by two-way ANOVA. **(B)** Superoxide Dismutase (SOD) activity was measured in fresh the heart of mice across treatment groups by colorimetric kit. Absorbance was detected in a microplate reader at 450 nm. Background absorbance was subtracted from all data points, including standards. All data: mean ± SEM (N = 3–5). *P < 0.05 vs. control and #P < 0.05 vs. Ang II by two-way ANOVA.

A previous study has demonstrated that oxidative stress can modulate the activity of BK channels, leading to changes in their function (Coskun and Tokgun, 2021). Superoxide dismutase (SOD) is an enzyme that plays a crucial role in the body’s antioxidant defense system and recent research has suggested a potential link between SOD and fibrosis (Kliment et al., 2009). To investigate the effect of BK channel activation on oxidative stress, reactive oxygen species (ROS) and SOD were examined in the heart of Ang II mice. We found that Ang II treatment significantly increased ROS production by 1.4-fold. Activation of the BK channel with BMS was effective in abolishing this change (Figure 3A). SOD production was 40% downregulated in the heart of Ang II mice group; treatment with BMS reversed this change (Figure 3B). These results suggest that activation of BK channels alleviated Ang II-induced oxidative stress.

### Activation of BKα attenuated ang II-induced macrophage infiltration and ang II-induced upregulation of inflammation in the heart

**FIGURE 4:**
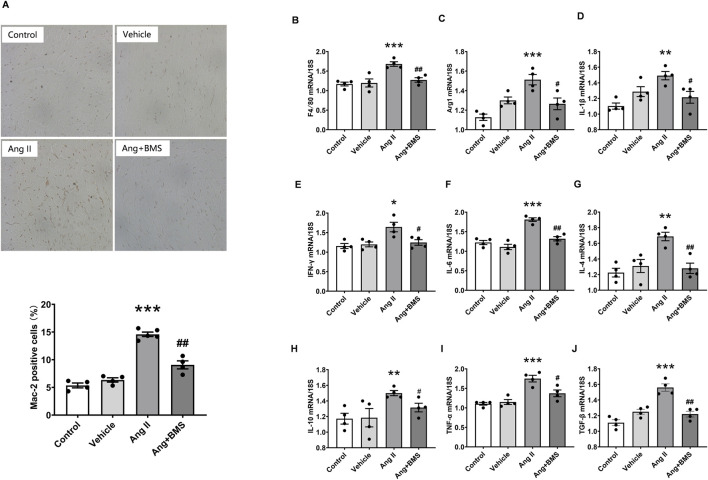
Activation of BKα attenuated Ang II-induced macrophage infiltration and AII-induced upregulation of inflammation in the heart. **(A)** Immunohistochemistry staining for macrophage (Mac-2) in mouse heart of control, vehicle, Ang II and Ang II plus BMS191011-treated mice. Images were captured at 200× magnification; scale bars = 50 μm. Quantification of Mac-2 was calculated as the percentage of positive area over the total tissue area using ImageJ analysis software. Data: mean ± SEM (N = 5). ***P < 0.05 vs. control and ##P < 0.05 vs. Ang II by two-way ANOVA. **(B–J)** The quantitative mRNA expression for F4/80 **(B)**, Arg1 **(C)**, IL-1β **(D)**, IFN-γ **(E)**, IL-6 **(F)**, IL-4 **(G)**, IL-10 **(H)**, TGF-β **(I)**, TNF-α **(J)** in mouse hearts were performed by real-time qPCR. Individual gene expressions were calculated by ΔΔcq and standardized by housekeeping gene 18s. All data: mean ± SEM (N = 4). *P < 0.05, **P < 0.01, ***P < 0.01 vs. control and #P < 0.05, ##P < 0.01 vs. Ang II by two-way ANOVA.

Mac-2, a galactose-binding protein secreted by activated macrophages, is commonly employed as an indicator of macrophage activation in response to various stimuli. (Sato and Hughes, 1994). To discover the effect of Ang II on macrophage secretion, we detected macrophages in the heart of all four groups of mice as described in methods. The number of mac-2 positive cells increased by 2.8-fold in Ang II mice compared with the control cohort. Activation of BKα successfully prevented this increase (Figure 4A). Additionally, macrophages are versatile immune cells that can polarize into different functional phenotypes based on the signals they receive from their microenvironment. The two primary states of polarization are M1 (pro-inflammatory) and M2 (anti-inflammatory) macrophages. We tested whether Ang II promoted macrophage polarization. F4/80, a macrophage-restricted cell surface glycoprotein, is commonly used to identify the total macrophage populations. IL-1β serves as a marker for M1 macrophages, while Arginase-1 is used as a marker for M2 macrophages. The expression of these markers was significantly increased in the heart tissue of Ang II-treated animals, with a 1.4-fold increase in F4/80, a 1.2-fold increase in IL-1β, and a 1.3-fold increase in Arginase-1 compared with vehicle treatment. Treatment with BKα opener attenuated these increases (Figures 4B–D).

Ang II is recognized as a powerful proinflammatory mediator (Chang and Wei, 2015). To investigate the relationship between BK channel activity and the levels of inflammation markers in heart tissue following Ang II treatment, the expression of inflammatory markers was evaluated by qPCR. Significant increases in the mRNA expression of the inflammatory factors: IFN-γ, IL-6, IL-4, IL-10, TNF-α, and TGF-β were observed in the Ang II group compared to the vehicle group. However, BMS191011 treatment effectively prevented the Ang II-induced upregulation of these markers (Figures 4E–J). These data suggest that Ang II induces macrophage infiltration and upregulation of inflammation, and that BK channel activation attenuates macrophage polarization in the heart tissue of cardiac Ang II mice, contributing to an anti-inflammatory effect.

### Activation of BKα attenuated ang II-induced polarization of macrophages *in vitro*

**FIGURE 5:**
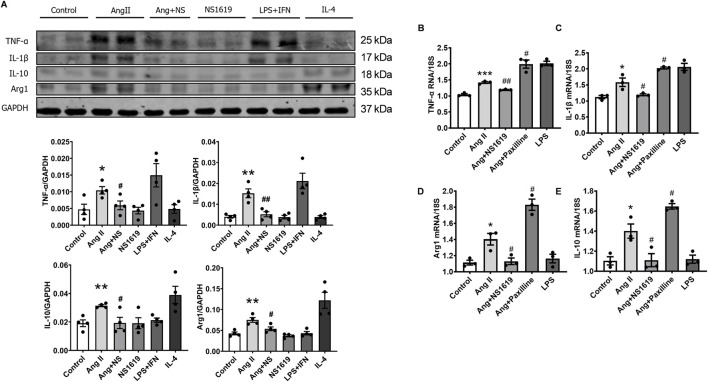
Activation of BKα attenuated Ang II-induced polarization of macrophages *in vitro*. **(A)** Cultured macrophages were treated with Ang II (100 nM), Ang II + NS1619 (20 μM), NS1619 (20 μM), LPS (100 ng/mL) + IFNγ (10 nM), and IL-4 (20 nM) for 24-h. The protein abundance of TNF-α, IL-1β, IL-10, and Arg1 was measured using Western blot. All Western blot data was normalized to GAPDH as control. Mean ± SEM (N = 5). *P < 0.05, **P < 0.01, vs. control and #P < 0.05 vs. Ang II by two-way ANOVA. **(B–E)** Macrophages were treated with Ang II, Ang II + NS1619, Ang II + Paxilline (10 μM), and LPS + IFN (10 nM) for 24-h. The mRNA expression of. **(B)** TNF-α. **(C)** IL-1β. **(D)** Arg1, and. **(E)** IL-10 were measured by real -time qPCR and normalized to 18 S rRNA. All data: mean ± SEM (N = 3–5). *P < 0.05, ***P < 0.01 vs. control and #P < 0.05 vs. Ang II by two-way ANOVA.

Since we found that Ang II promoted macrophage infiltration in the heart of Ang II mice, we further planned to discover the connection between BK channel activity and macrophage polarization. The activation markers of M1 macrophage (IL-1β, TNF-α) and M2 macrophage proteins (IL-10 and Arg-1) were evaluated in cultured macrophages (RAW 264.7 TIB-71) with Ang II and/or BK opener NS-1619 treatment. LPS/IFNγ was used for the positive control for the activation of M1 and IL-4 was used for activation of M2. Treatment of macrophages with 100 nM Ang II for 24 h significantly increased IL-1β, TNF-α, IL-10 and Arg-1, while treatment with NS-1619 was effective in reducing Ang II-induced upregulation (Figure 5). The mRNA expression of IL-1β (1.4-fold), TNF-α (1.4-fold) IL-10 (1.3-fold) and Arg-1 (1.3-fold) increased in Ang II treated cells and was returned to normal ranges after administration of NS-1619 (Figures 5B–E). When paxilline, a highly selective BK inhibitor, was added with Ang II, there was an additional increase in these macrophage markers (Figures 5B–E). These data suggest that the activation of BK channel attenuates the polarization of macrophages after Ang II treatment, and the inhibition of BK channels has the opposite impact.

### Activation of BKα attenuated Ang II-induced upregulation of the NF-κB-NLRP3 signaling pathway in cultured macrophages and in the heart of hypertensive mice

**FIGURE 6:**
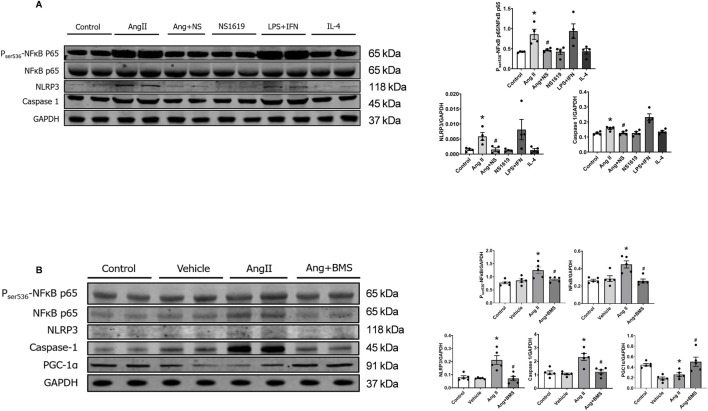
Activation of BKα attenuated Ang II-induced upregulation of NF-κB signaling in cultured macrophages and in the heart of Ang II treated mice. **(A)** Cultured macrophages were treated with Ang II (100 nM), Ang II + NS1619 (20 μM), NS1619 (20 μM), LPS (100 ng/mL) + IFN (10 nM), and IL-4 (20 nM) for 24-h. For each group, protein abundance of Pser536-NFκB p65, NFκB p65, NLRP3, and caspase-1 was measured using Western blot. All Western blot data was normalized to GAPDH as control. All data: Mean ± SEM (N = 4). *P < 0.05 vs. control and #P < 0.05 vs. Ang II by two-way ANOVA. **(B)** Protein abundance of pser-536-*NF-κB* p65, *NF-κB* p65, NLRP3, PGC-1α, and caspase-1 were measured by Western blot in the heart of control, vehicle, Ang I, and Ang II plus BMS191011-treated mice. The data was normalized to GAPDH. Mean ± SEM (N = 5). *P < 0.05 vs. control and #P < 0.05 vs. Ang II by two-way ANOVA.

Studies have revealed that persistent overactivation of renin-angiotensin system (RAS) results in a concomitant upregulation of nuclear factor NF-κB (Nagai et al., 2007). To explore whether a BK channel opener impacts NF-κB signaling, we evaluated the protein levels of NF-κB, NLRP3 and caspase-1 in Ang II-treated macrophage with or without BK opener NS-1619 intervention. Significant increases in NF-κB phosphorylation (calculated by the ratio of serine-536 phospho- NF-κB to NF-κB) was seen in the Ang II group compared to the control, while treatment with NS-1619 blocked this change (Figure 6A). Again, LPS/IFN-γ was used as positive control for the activation of M1, and this treatment also significantly enhanced NF-κB phosphorylation. IL-4 did not change the NF-κB phosphorylation amount, indicating that M2 activation did not impact NF-κB phosphorylation. We also observed that NLRP3 and caspase 1, two downstream NF-κB-induced signaling proteins, were also increased by ang II and LPS/IFN-γ compared with the control group. These data suggest that the attenuation of the inflammation and/or polarization of macrophages under Ang II treatment by BK channel activation could be mediated through inhibition of NF-κB signaling.

We also evaluated the NF-κB-NLRP3 induced proteins in the hearts of Ang II-treated mice. A significant increase in the expression of NF-κB and Ser-536 phosphorylation were seen in the Ang II cohorts compared to the vehicles and controls. The NF-κB downstream proteins NLPR3 and caspase one were also significantly increased in the Ang II heart. Treatment with BMS-191011 effectively eliminated these changes (Figure 6B).

Peroxisome proliferator-activated receptor gamma coactivator 1-alpha (PGC-1α) is a key regulator of mitochondrial biogenesis. The amount of PGC1α was significantly decreased in the heart of Ang II mice, and treatment with the BK opener reduced this effect (Figure 6B). These data suggest that the activation of BK channel limited Ang II induced upregulation of NF-κB-NLRP3 signaling and downregulation of PGC1α in the heart tissue of hypertensive mice.

## Discussion

Hypertension remains a leading cause of cardiovascular morbidity and mortality worldwide, and its associated complications, including cardiac fibrosis, pose significant therapeutic challenges (Whelton et al., 2018). Hypertension induced by angiotensin II drives cardiac remodeling, including oxidative stress, inflammation, and activation of profibrotic pathways such as TGF-β/Smad. Our study using Ang II induced hypertension models, provides compelling evidence that activation of BK channels mitigates Ang II-induced hypertension and cardiac fibrosis through multiple interlinked mechanisms including modulation of the NFκB-NLRP3 signaling pathway. These findings highlight the therapeutic potential of BK channel activators in the management of hypertensive cardiac damage.

While recent advances focus on delivering supportive biomaterial scaffolds to improve cell retention and signaling, our work demonstrates that pharmacologically targeting an endogenous ion channel (BK) can similarly ameliorate a hostile myocardial microenvironment by suppressing key inflammatory and fibrotic pathways (Yao et al., 2026). Notably, the regulatory relationship between BK channel activity and the NF-κB/NLRP3 inflammatory axis has been reported in other organ systems and fibrotic disease models, including our previous work (Wang et al., 2022b) and recent studies (Zyrianova et al., 2025). However, the present study extends these observations to Ang II-induced hypertensive cardiac injury and provides *in vivo* evidence that BK activation coordinately modulates inflammation, oxidative stress, and macrophage polarization within the cardiac microenvironment.

Our data shows that BK activation suppresses an Ang II-induced increase in NF-κB phosphorylation and reduces expression of NLRP3 and caspase-1 in macrophages, as well as in cardiac tissue. Phosphorylation of NF-κB (e.g., at Ser536) enhances the activity of NF-κB. Activated NF-κB translocates to the nucleus and transcriptionally regulates inflammatory responses via binding to the promoter region of a variety of pro-inflammatory genes, such as NLRP3, IL-1β, IL-6, CXCL8 (IL-8), pro-IL-1β and caspase-1 (Vogel and Matsumura, 2009). Escobar et al. reported that stimulation of the BK channel with NS-11021 reduced IL-1β production in macrophages, opposing the effect of BK inhibition (Chen et al., 2024a). This supports a model where BK activity negatively regulates inflammasome signaling. Our findings extend that concept to Ang II mediated hypertension *in vivo* and *in vitro*.

Although Ang II reduced BK channel activity in the HEK293/BKα system, it should be noted that the concentration used *in vitro* was higher than physiological levels. At such concentrations, Ang II may induce additional cellular stress responses beyond classical AT_1_ receptor–dependent signaling. Therefore, while these experiments demonstrate that BK activity can be suppressed under strong Ang II stimulation, the precise upstream mechanisms linking Ang II to BK inhibition *in vivo* likely involve receptor-mediated and post-translational regulatory pathways that warrant further investigation.

Chronic inflammation plays a pivotal role in the progression of hypertensive cardiac remodeling (Chang and Wei, 2015). Our results reveal that Ang II induces a significant upregulation of inflammatory markers, including IL-4, IL-10, IL-6, TNF-α, IFN-γ and IL-1β, which was markedly attenuated by BK activation. These findings suggest that BK channel modulation exerts an anti-inflammatory effect, potentially through its influence on macrophage polarization. Ang II promoted macrophage polarization of macrophages towards M1 phenotype, whereas BK activation blocked this shift (Yang et al., 2020). This observation is consistent with prior evidence demonstrating that BK channel activation reduces macrophage-induced inflammation in renal fibrosis models (Wang et al., 2022b). Our studies in the cardiovascular system suggest that targeting BK channels may provide a novel strategy to regulate immune responses in hypertensive heart disease. Interestingly, while M2 macrophages are generally considered anti-inflammatory and associated with tissue repair, their prolonged activation in the context of Ang II stimulation can paradoxically contribute to fibrosis. M2 macrophages secrete profibrotic factors, including TGF-β and IL-10, which could drive the trans-differentiation of cardiomyoblasts to myofibroblasts and excessive extracellular matrix deposition.

There is growing evidence that TGF-β1 decreases BK activity, and BK channels can influence TGF-β–driven fibrotic responses. Manzanares et al. found that TGF-β1 decreased BK activity by downregulating BK subunit expression and function, producing an ion-transport effect mediated by TGF/Smad signaling in cultured bronchial epithelia cells (Manzanares et al., 2015). Indeed, we found that TGF-β was increased and BK channel activity decreased in our cardiac fibrosis mice. In addition, we observed that Ang II markedly upregulated TGF-β along with the fibrosis markers fibronectin, vimentin, and α-SMA, while BK activation suppressed these changes. These results are in line with previous findings by us and others (Wang et al., 2022b; Wang et al., 2019). Additionally, in a liver fibrosis rat model, overexpression of the BK pore-forming α-subunit (KCNMA1) or pharmacologic activation of BK channels suppressed TGF-β1/Smad3 signaling and inhibited liver fibrosis (Yang et al., 2020). In kidney fibrosis models, another study reported that BK activation decreased TGF-β/Smad2/3 signaling by accelerating the degradation of TGF-β receptors via caveolae‐mediated processes (Wang et al., 2022b). Furthermore, the anti-fibrotic role of BK channels may be linked to their ability to modulate calcium homeostasis and ion channel regulation, thereby preventing myofibroblast activation (Xing et al., 2023; Scruggs et al., 2020).

Oxidative stress is a key contributor to hypertension-induced cardiac damage (Liu and Desai, 2015). Recent advances in redox biomarker detection further highlight the pathological relevance of mitochondrial oxidative stress in cardiovascular diseases; for example, an electrochemical biosensor targeting mitochondrial Complex I glutathionylation has been proposed as a sensitive indicator of myocardial infarction risk, suggesting that future studies could more precisely evaluate BK-mediated regulation of mitochondrial redox states (H et al., 2026). The increase in oxidative stress could be due to Ang II-induced downregulation of PGC1α, since we found that PGC1α protein is significantly decreased along with increased ROS production and decreased SOD in Ang II heart. Provision of BK opener abolished these changes. Our observations are consistent with the study of the PGC-1α deficiency mice, which produced reduced mitochondrial antioxidant enzymes (including SOD), aggravated oxidative stress, and worsened left ventricular dysfunction (Lu et al., 2010). In addition, PGC-1α not only drives mitochondrial antioxidant gene expression but also suppresses NF-κB activation, balancing redox and inflammatory responses (Huang et al., 2024). Thus, in our model, BK activation likely rescues redox balance, possibly via upregulation of PGC-1α or downstream effectors, breaking the feed-forward loop between oxidative stress and inflammation. In addition, BK channels are known to localize not only on the plasma membrane but also in mitochondria (mitoBK), where they play a critical role in regulating mitochondrial ROS production and inflammatory signaling. Although our study did not specifically distinguish between plasma membrane and mitochondrial BK activation, the observed restoration of PGC-1α expression, reduction in ROS, and suppression of NF-κB/NLRP3 signaling suggest a potential involvement of mitochondrial BK-mediated regulation of mitochondrial inflammatory tone. Given that macrophage inflammasome activation is highly dependent on mitochondrial dysfunction and redox imbalance, it is plausible that pharmacological BK activation by BMS-191011 or NS-1619 exerts cardioprotective effects, at least in part, through modulation of mitoBK-associated mitochondrial signaling pathways. Future studies using mitoBK-selective approaches will be required to further dissect the subcellular specificity of BK channel actions. In agreement with our study, Chen et al. showed that oxidative stress drives fibrosis via TGF-β1/MAPK signaling pathway (Chen et al., 2024b). Kampa also reported that BK channel activation has cardioprotective effects by effectively restoring SOD levels and reducing oxidative stress (Kampa et al., 2022).

It should also be noted that the BK-dependence of the *in vivo* effects was inferred primarily from pharmacological activation and *in vitro* blockade experiments using paxilline. We did not use *in vivo* BK channel inhibition or genetic loss-of-function models, and therefore the observed improvements in blood pressure and cardiac fibrosis cannot be attributed exclusively to BK channels. Considering that pharmacological BK activators such as BMS-191011 and NS-1619 may exert off-target effects, especially at higher concentrations, our findings should be interpreted as indicating a predominant, but not necessarily exclusive, role of BK channel activation in mediating the cardioprotective effects.

Taken together, our findings position BK channels as nodal regulators that converge on multiple pathogenic axes: inflammation (NF-κB/NLRP3), oxidative stress (SOD, likely PGC-1α mediated), and profibrotic signaling (TGF-β/Smad). Notably, this integrative protective pattern is conceptually consistent with other receptor-mediated anti-remodeling pathways; for instance, activation of the Mas-related G-protein–coupled receptor MrgD was recently shown to attenuate pulmonary hypertension and fibrosis via inhibition of the AKT–MAZ–PIM1 axis, suggesting that ion channels and GPCR signaling may share convergent downstream mechanisms in combating cardiovascular stress (Zhong et al., 2025). By influencing macrophage behavior, BK activation appears to cut off upstream sources of both inflammatory cytokines and TGF-β. This multifaceted protective effect suggests therapeutic potential of BK activators in hypertensive heart disease, beyond their known vascular effects.

In summary, we demonstrate that in Ang II-induced hypertensive mice, BKα expression declines in the heart and activation of BK channels reduces hypertension, cardiac fibrosis, oxidative stress, macrophage infiltration/polarization, and NF-κB/NLRP3 inflammasome activation. These effects are accompanied by suppression of TGF-β/Smad signaling and restoration of antioxidant defenses likely linked to PGC-1α. Together, our results add to growing literature supporting pharmacological activation of BK channels as a promising therapeutic strategy for hypertensive heart disease.

## Data Availability

The raw data supporting the conclusions of this article will be made available by the authors, without undue reservation.
