# Supplementary material for: Activation of BK channels ameliorates cardiac injury Via NFκB-NLRP3 signaling in angiotensin II-induced hypertension mouse model
Source: Front Pharmacol. 2026 Jun 15;17:1739677. doi: 10.3389/fphar.2026.1739677 (PMC13310761; doi:10.3389/fphar.2026.1739677)
Supplement: Supplementary file 1 [file Table1.docx]

Supplementary Table 1. Primers Sequences

| IL 6-M-F | TTCCATCCAGTTGCCTTCTTG |
| --- | --- |
| IL 6-M-R | TTGGGAGTGGTATCCTCTGTGA |
| IL10-M-F | CCAAGCCTTATCGGAAATGATC |
| IL10-M-R | CACCCAGGGAATTCAAATGC |
| TNF-α-M-F | GACGTGGAACTGGCAGAAGAG |
| TNF-α-M-R | GCCACAAGCAGGAATGAGAAG |
| Arg1-M-F | AACACGGCAGTGGCTTTAAC |
| Arg1-M-R | GAGGAGAAGGCGTTTGCTTA |
| IL-1β-M-F | CTTCCCCAGGGCATGTTAAG |
| IL-1β-M-R | ACCCTGAGCGACCTGTCTTG |
| IL 4-M-F | GGAGATGGATGTGCCAAACG |
| IL 4-M-R | CGAGCTCACTCTCTGTGGTGTT |
| TGF-β-M-F | GACTCTCCACCTGCAAGACCAT |
| TGF-β-M-R | GGGACTGGCGAGCCTTAGTT |
| IFN-γ-M-F | TGCTGATGGGAGGAGATGTCT |
| IFN-γ-M-R | TTTCTTTCAGGGACAGCCTGTT |
| F4/80-M-F | CTTTGGCTATGGGCTTCCAGTC |
| F4/80-M-R | GCAAGGAGGACAGAGTTTATCGTG |
| 18S-F | CCAGAGCGAAAGCATTTGCCAAGA |
| 18S-R | TCGGCATCGTTTATGGTCGGAACT |
